# Cognition and mental wellbeing after electrical accidents: a survey and a clinical study among Swedish male electricians

**DOI:** 10.1007/s00420-020-01520-x

**Published:** 2020-02-08

**Authors:** Sara Thomée, Kai Österberg, Lisa Rådman, Kristina Jakobsson

**Affiliations:** 1 Department of Psychology, University of Gothenburg, P.O. Box 500, 405 30 Gothenburg, Sweden; 2 Department of Psychology, Lund University, P.O. Box 213, 221 00 Lund, Sweden; 3 Department of Occupational and Environmental Medicine, Örebro University Hospital, 701 85 Örebro, Sweden; 4 Department of Physiotherapy, School of Medical Sciences, Örebro University, 701 82 Örebro, Sweden; 5 School of Public Health and Community Medicine, Institute of Medicine, University of Gothenburg, P.O. Box 414, 405 30 Gothenburg, Sweden; 6 Occupational and Environmental Medicine, Sahlgrenska University Hospital, P.O. Box 414, 405 30 Gothenburg, Sweden

**Keywords:** Occupational injury, Occupational accident, Neuropsychology, Psychological health, Electrical injury, Electrical accident, Symptoms

## Abstract

**Purpose:**

The purpose was to examine long-term consequences of exposure to electrical current passing through the body. We investigated (1) whether electricians after having experienced an electrical accident report more cognitive problems and lower mental wellbeing and (2) have objectively verifiable reduced cognitive function; and (3) which circumstances at the time of the accident affect long-term subjective cognitive function and mental wellbeing?

**Methods:**

A survey of male electricians who had experienced electrical accidents (*n* = 510) and a clinical study in a subsample (*n* = 23) who reported residual health problems was carried out. Both groups were examined regarding subjective cognitive function (Euroquest-9) and mental wellbeing (Symptom Checklist-90 subscales). The clinical study included neuropsychological tests of memory, attention, spatial function, and premorbid intellectual capacity. A matched control group was retrieved from reference data.

**Results:**

The survey participants reported more cognitive problems and lower mental wellbeing than referents. Of the examined circumstances, having experienced mortal fear at the time of the accident and health complaints, especially mental symptoms, for > 1 week after the accident were the most significant risk factors for later subjective cognitive problems and lower mental wellbeing. The only statistically significant difference in neuropsychological tests was better performance in part of the memory tests by the clinical study group compared to the control group.

**Conclusions:**

The participants reported more cognitive problems and lower mental wellbeing than referents, but no long-term objective cognitive dysfunction was detected. Emotional response at the time of the accident and health complaints in the aftermath of the accident may constitute important indications for medical and psychological follow-ups.

**Electronic supplementary material:**

The online version of this article (10.1007/s00420-020-01520-x) contains supplementary material, which is available to authorized users.

## Introduction

Electrical accidents involving electrical current passing through the body have been reported to generate long-term physical consequences in terms of pain and neurological symptoms, such as numbness and sensory disorders, and, for example, hearing loss (Bailey et al. 2008; Fish et al. 2012; Kærgaard 2009; Rådman et al. 2016a, b; Singerman et al. 2008; Wesner and Hickie 2013). Moreover, long-term cognitive and psychiatric disorders after electrical injury (EI) have been reported (Bailey et al. 2008; Barrash et al. 1996; Duff and McCaffrey 2001; Piotrowski et al. 2014; Pliskin et al. 1998, 2006; Ramati et al. 2009; Singerman et al. 2008), predominantly in patient populations. For example, in a study by Pliskin et al. (1998), EI patients reported cognitive problems more often than matched healthy controls, with almost half of the participants reporting problems with concentration, difficulties finding words, and slower mental speed. In one study (Pliskin et al. 2006), EI patients performed poorer in neuropsychological examinations than matched healthy controls on measures of attention and mental and motor speed. Duff and McCaffrey (2001) reviewed eight studies of neuropsychological effects after EI and the findings indicated reduced short-term memory, attention, concentration, sensory motor skills, and visuospatial ability. Primeau et al. (1995) and Barrash et al. (1996) point out that the cognitive complaints after EI resemble those in mild to moderate traumatic brain injury. However, it seems that the level of problems after EI can be hard to predict (Andrews 2012; Primeau et al. 1995) and EI symptoms may occur or worsen after a delay (Bailey et al. 2008). In the previously mentioned study by Pliskin et al. (1998), the self-reported cognitive symptoms were more pronounced in the post-acute phase than in the acute phase after the accident.

Being the victim of an occupational (or any) accident can be traumatic and lead to increased psychological problems (Chin et al. 2017; Lin et al. 2014) and this is evidently the case also for electrical accidents (Kelley et al. 1999; Piotrowski et al. 2014; Pliskin et al. 1998; Ramati et al. 2009). Andrews (2012) indicates that psychological disability may be the biggest problem for an EI patient. In Pliskin et al. (1998), approximately half of the EI patients reported anxiety and depressed mood. Ramati et al. (2009) performed a psychiatric evaluation of 86 EI patients and found that 78% warranted a psychiatric diagnosis, such as depression or posttraumatic stress disorder (PTSD). Psychiatric problems seemed to increase with time, and were not dependent on, for example, voltage level or chronic pain (Ramati et al. 2009). One of the risk factors for PTSD and major depression after EI, as reported in a retrospective study of 73 EI patients (Kelley et al. 1999), was having experienced the no-let-go phenomenon, i.e., when muscle contractions caused by the current give an involuntary grip and thus prolong exposure. The no-let-go phenomenon emerged as a particularly stressful event also in our interview study regarding electricians’ experiences of an accident (Thomée and Jakobsson 2018). The electricians described severe anxiety and mortal fear in a situation where they could not voluntarily release from the electrical current (Thomée and Jakobsson 2018). Other risk factors named by Kelley et al. (1999) included having experienced altered consciousness or loss of consciousness at the time of the accident.

Most previous studies of cognitive or emotional problems after electrical accidents have involved patient populations. The approach of this study was instead to include electricians who had been exposed to electrical current passing through the body but who had not all sought medical care as a result. The study was part of a research project done in collaboration between several occupational medicine clinics in Sweden which had the aim to examine a wide range of long-term effects of EI. The incentive was to increase knowledge as a basis for improved management of EI, both in the health care setting and at the workplace, as well as for diagnosis, treatment, and prevention of long-term physical and psychological dysfunction (Rådman et al. 2016a, b; Ek et al. 2015; Thomée and Jakobsson 2018; Tondel et al. 2016). The focus of this particular study was on long-term consequences for cognition and mental wellbeing after EI, and it includes results from a survey and a clinical study.

### Aim

The aim of this study was to examine whether exposure to electrical current passing through the body has long-term consequences for cognition and mental wellbeing.

Specific research questions were:

1. Does exposure to electrical current have long-term consequences for a person’s subjective cognitive function and mental wellbeing?
2. Can reduced cognitive function be objectively verified?
3. How do circumstances at the time of the accident affect long-term subjective cognitive function and mental wellbeing?

## Methods

The study consisted of two parts: a survey study of 510 male electricians who had experienced at least one incident involving electrical current passing through the body, and a clinical study with neuropsychological tests in a subgroup of 23 electricians who had reported residual health complaints after an electrical accident.

### Participants

#### Survey study (*n* = 510)

The survey study participants were recruited from two sources, the Swedish Electricians’ Union (SEU) and the Swedish Work Environment Authority (SWEA) registry of work-related injury. The target population in the SEU group was male electricians born between 1946 and 1993, who were members of the installation, service, or power plant sectors of the union, and who resided in the counties associated with the Swedish regional occupational medicine clinics in Gothenburg, Lund, Sundsvall, Umeå, and Örebro. Women were not included because they only constituted about 1% of the active members of the SEU at the time of the study. From approximately 12,000 eligible members, a random sample of 4000 was selected. In addition, 343 persons who had reported EI to the SWEA registry in 2004–2011 and who resided in the same geographical area were identified. For reasons of confidentiality, administration of the survey to the eligible participants from the SWEA registry was handled by SWEA personnel. The SWEA registry is not limited to professional electricians and women also received the questionnaire.

**Fig. 1:**
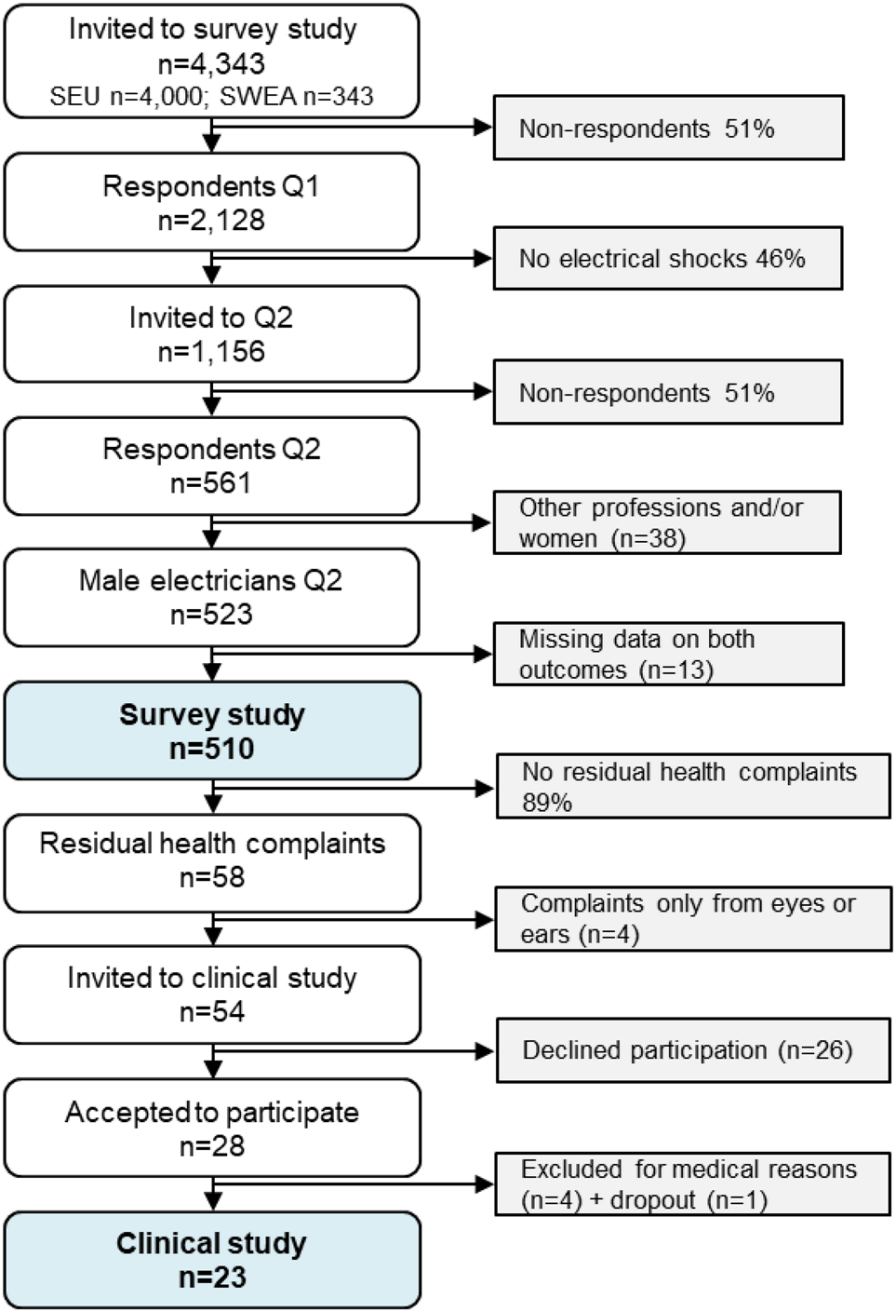
Study participation process. Q1 = Questionnaire 1; Q2 = Questionnaire 2

Consequently, 4343 individuals were sent a first survey in September–October 2011 with questions about occupational electrical accidents and perceived residual health effects. After two reminders to the SEU group and one reminder to the SWEA group, the response rate was 49% (*n* = 2128). A second questionnaire was sent in March 2012 to the 1156 individuals (including 34 women) who indicated that they had experienced at least one occupational accident resulting in electrical current passing through their body in the past 5 years. This second questionnaire contained questions about the respondents’ most severe incident of electrical current passing through the body, including perceived health effects. After two reminders, 561 respondents (49%) answered the questionnaire. After exclusion of female respondents (*n* = 18) and males with other professions (*n* = 20) from the SWEA group, there were 523 male professional electrician respondents. For the current study, those with missing data on both outcome measures regarding subjective cognition and mental wellbeing were excluded, leaving 510 in the survey study group (Fig. 1).

#### Clinical study (*n* = 23)

The target population for the clinical study was respondents in the survey study group who in the second questionnaire had reported residual health complaints (i.e., their health complaints had decreased, increased, or were unchanged) that they attributed to EI (*n* = 58). After excluding four persons who reported symptoms in only the eyes or ears, 54 persons were invited to participate in a clinical study involving neurophysiological and neuropsychological examinations and interviews. Twenty-eight agreed to participate, four of whom were excluded due to medical reasons (neurological or cardiovascular disease, or other severe conditions). One potential participant dropped out of the study. In total, 23 participants completed the clinical study (Fig. 1).

### Measurements in the survey study

Data from the survey study questionnaire included year of birth, highest level of completed education (UNESCO-UIS 2006), and number of severe incidents that involved an electrical current passing through the body.

#### Circumstances of the accident

The survey contained questions regarding the perceived most severe incident: year and month of the accident; voltage level (< 110 V, 110 V, 220/230 V, 231–400 V, 401–1000 V, or > 1000 V); points of contact (hand/finger, arm, head, body, leg, foot, uncertain); current pathway (unilateral/bilateral), having experienced the no-let-go phenomenon (yes/no); having felt dazed afterwards (yes/no/uncertain); and having lost consciousness (yes/no/uncertain). In the regression analyses, the response “uncertain” was set as “missing.” Low voltage was defined as ≤ 1000 V and high voltage as > 1000 V.

#### Emotional responses at the time of the accident

The participants were asked to mark if they had felt: (1) incapacitated; (2) mortal fear; and/or (3) rage at the time of the accident (yes/no/uncertain). In the regression analyses, the response “uncertain” was set as “missing.”

#### Health complaints after the accident

The participants were asked to indicate if, following the accident, they had had health complaints that they attributed to the exposure to electrical current. The list of potential complaints included eyes, hearing, tinnitus, pain, muscle weakness, muscle twitching, loss of sensation, memory problems, concentration difficulties, sleep problems, anxiety, and fatigue. Response categories for each were 1 = had no complaints; 2 = had complaints for < 1 week after the incident; 3 = had complaints for > 1 week after the incident. Participants whose responses included response category 3 were categorized as “Health complaints > 1 week.” Participants whose responses included response category 2 (and no category 3) were categorized as “Health complaints < 1 week.” Participants who had responded with category 1 answer to at least one symptom (and no category 2 or 3) were categorized as “No health complaints”. The variable Health complaints were also divided into Physical symptoms (eyes, hearing, tinnitus, pain, muscle weakness, muscle twitching, loss of sensation) and Mental symptoms (memory problems, concentration difficulties, sleep problems, anxiety, and fatigue). The two variables overlap in that respondents can report both physical and mental symptoms. Only 5 persons reported having only mental health complaints after the accident.

In addition, the participants were asked if they still had complaints from the same list. The response categories were 1 = complaints have disappeared; 2 = complaints have decreased; 3 = complaints are unchanged; and 4 = complaints have increased. Response categories 2–4 were categorized as “Residual health complaints”, and response category 1 or no response as “No residual health complaints”.

#### Subjective cognitive problems

The Euroquest-9 (EQ-9), i.e., the memory and concentration scale from the Euroquest (EQ) questionnaire (Chouanière et al. 1997) was included in the survey questionnaire to screen for subjective cognitive problems. The EQ-9 has been validated as a sensitive measure of cognitive impairment (Carter et al. 2002; Chouanière et al. 1997; Karlson et al. 2000). It contains six questions regarding memory and three questions regarding attention and concentration. Responses are given on a 4-point scale, from 1 = “rarely/never” to 4 = “very often.” The full EQ-9 scale was used, but it was also divided into two subscales, EQ Memory and EQ Attention. Mean scores in the subscales were calculated.

#### Mental wellbeing

Three subscales from the Symptom Checklist-90 (SCL-90) (Derogatis 1992) were included in the survey questionnaire to measure mental wellbeing. The subscales were Somatization (12 items), Depression (13 items), and Anxiety (10 items). Responses were given on a 5-point scale, from 0 = “not at all” to 4 = “very much.” Mean values for each subscale were calculated.

#### Reference data for the EQ-9 and SCL subscales

Reference data for the EQ-9 and SCL-subscales were obtained from a group of healthy male volunteers with similar demographics, who had participated in a previous study using the same scales and similar neuropsychological tests (*n* = 98) (Persson et al. 2002).

### Measurements in the clinical study

#### Subjective cognitive problems

Data from the EQ-9 (described in “Subjective cognitive problems”) were collected from the survey study, for the clinical study participants.

#### Mental wellbeing

Data from the SCL subscales (described in “Mental wellbeing”) was collected from the survey study, for the clinical study participants.

#### Cognitive test battery

The selected set of tests covered memory functions [Austin Maze test with the Milner pathway (Walsh 1985) and Cronholm-Molander Word Pairs (Cronholm and Molander 1957)]; attention/working memory [WAIS-R Digit Symbol including incidental learning (Wechsler 1992), d2 Test of Attention (Brickenkamp and Zillmer 1998), and F-A-S verbal fluency test (Benton et al. 1994)]; visuospatial function [WAIS-R Block design (Wechsler 1992)]; and premorbid intellectual performance [SRB:1 Synonyms test (Dureman et al. 1971) and WAIS-R Information (Wechsler 1992)]. Raw data from the tests were used. The tests were chosen because they have been found to be sensitive to subtle encephalopathy in previous studies (Österberg et al. 2000), or have been used for studying the effects of EI elsewhere (Duff and McCaffrey 2001), and because we largely had access to reference data for the tests (Persson et al. 2002).

#### Procedures of the clinical study

The clinical study participants were examined at the occupational medicine clinics in Gothenburg, Lund, Sundsvall, Umeå, and Örebro in August–October 2012, approximately 6 months after the survey. A psychologist (first author S.T.) administered the cognitive tests and scales. In total, the psychological investigation, which also included an interview (Thomée and Jakobsson 2018), took 3–4 h. On the same day, the participants underwent a neurophysiological examination (2.5–3 h) conducted by a physiotherapist (Rådman et al. 2016a). These two investigations alternated so that half of the participants started with the psychological examination and half with the neurophysiological investigations. In total, the participants spent 6–7 h at the clinic including breaks.

#### Matched control group

A control group (*n* = 23) was retrieved from the previously mentioned reference sample of healthy male volunteers (Persson et al. 2002), who had participated in a previous study using similar neuropsychological tests and scales. Each participant in the clinical study group was closely matched with a healthy control with regard to gender, age, education (UNESCO-UIS 2006), and occupation (“technical work”). This procedure generated similar demographic distribution across the groups. Mean age of the matched controls was 46.8 [standard deviation (SD) 12.4, range 25–63] years. All but one of the matched controls worked in a technical field. For a few tests, data could not be obtained from the control group. For the Incidental learning part of the WAIS-R Digit Symbol, data from another healthy reference group (*n* = 50) (Österberg et al. 2014) were used, and for the d2 test of attention only official normative data were used. However, in contrast to the study group, these latter groups contained both men and women, and in the external reference group (*n* = 50) most were university-educated.

### Statistical analysis

All analyses for the survey study were carried out using SAS Enterprise 7.1 (SAS Institute, Cary, NC, USA). Comparisons of the clinical study group with the matched controls were carried out with IBM SPSS Statistics, version 20 (SPSS, Inc., Chicago, IL, USA).

Descriptive statistics, such as means and SDs for the continuous variables, and frequencies and percentages for the categorical variables, were calculated with PROC UNIVARIATE and PROC FREQ, respectively (SAS Institute, Cary, NC, USA). In the survey study, the six outcomes of the EQ-9 and SCL-subscales were compared to reference data using *t *tests (PROC TTEST). Further, linear regression models (PROC GENMOD) were used to analyze the independent variables, i.e., demographics, circumstances at the time of the accident, emotional responses, and health complaints after the accident, in relation to the six outcomes. The independent variables were checked for collinearity (Spearman’s correlations > 0.70). No collinearity was found; the highest correlation (*r* = 0.56) was between age and time since the accident. In the first step, univariate (crude) analyses were carried out for each independent variable in relation to the six outcomes. In the next step (Model I), age and time since the accident were added as covariates. In a second model, health complaints were added to the multivariate analyses.

Supplementary descriptive statistics were calculated with stratification for voltage (high and low). In addition, the low voltage group was analyzed separately using the same linear regression model as in the total survey group. The results are shown in supplementary tables.

In the clinical study, we used Mann–Whitney *U* test for variables that significantly deviated from the normal distribution (Shapiro–Wilk’s test, *p* < 0.05). Otherwise, *t *tests for independent groups were carried out to examine group differences between the clinical study participants and the matched controls.

The statistical significance level was set at *p* < 0.05.

## Results

### Survey study

#### Descriptives

The survey study group (*n* = 510) contained males aged 20–68 years (M 43.5, SD 13.2), see Table 1. The majority had completed secondary education (in many cases, vocational school). The self-reported number of severe incidents involving electrical current passing through the body during work varied from 0 to 90, with a median of 2. The subjectively perceived most severe incident, which was the focus of subsequent questions, had occurred a median of almost 7 years prior to the survey, ranging from within the month of the survey to almost 45 years previously. The incident had involved high voltage (> 1000 V) for 4% of the respondents. The most common contact point was the hand or finger. For 51% of the respondents the current pathway had been bilateral, i.e., had passed from one side of the body to the other. The head had been a contact point for 14 participants (3%) (data not shown). Twenty-six percent of respondents had experienced the no-let-go phenomenon. The majority (63%) had felt dazed after the accident, and 6% had lost consciousness.

**Table 1:** Descriptive statistics of the survey study group (*n* = 510) and the clinical study subgroup (*n* = 23)

	Survey study *n* = 510		Clinical study *n* = 23	
Age, years				
Mean (SD)	43.5 (13.2)		48.1 (13.8)	
Median	43		54	
Range	20–68		25–68	
No. of severe electrical shocks (*n* = 473; *n* = 23)				
Mean (SD)	3.4 (6.3)		4.3 (6.3)	
Median	2.0		1.0	
Range	0–90		1–25	
No. of years since the most severe accident (*n* = 374; *n* = 22)				
Mean (SD)	11.0 (10.5)		8.8 (10.3)	
Median	6.8		5.5	
Range	0–44.7		0.4–43.6	

	*n*	%	*n*	%
Education level (ISCED 1997)				
Primary school (level 2)	41	8	1	4
Secondary school (level 3)	429	85	20	87
University/college (level 5)	32	6	2	9
Circumstances of the most severe incident				
Voltage				
≤ 1000 V	420	96	16	73
> 1000 V	18	4	6	27
Electrical current contact points				
Hand—hand	223	51	14	61
Hand—other/unknown	189	44	7	30
Other—other/unknown	22	5	2	9
Current pathway				
Bilateral	169	51	12	60
Unilateral	164	49	8	40
No-let-go				
Yes	116	26	8	35
No	327	74	15	65
Loss of consciousness				
Yes	25	6	8	36
No	388	92	13	59
Uncertain	10	2	1	5
Dazed				
Yes	272	63	18	82
No	147	34	3	14
Uncertain	15	3	1	5
Emotional response at the time of the accident				
Incapacitated				
Yes	121	28	11	52
No	297	68	7	33
Uncertain	17	4	3	14
Mortal fear				
Yes	24	6	2	10
No	393	92	14	67
Uncertain	10	2	5	24
Rage				
Yes	193	45	7	30
No	222	51	13	57
Uncertain	18	4	3	13
Health complaints attributed to electrical injury				
Yes, > 1 week after the accident	66	15	20	87
Yes, < 1 week after the accident	90	20	3	13
No	286	65	–	–
Physical symptoms				
Yes, > 1 week after the accident	55	12	19	83
Yes, < 1 week after the accident	92	21	3	13
No	295	67	1	4
Mental symptoms				
Yes, > 1 week after the accident	25	6	6	30
Yes, < 1 week after the accident	46	10	5	25
No	368	84	9	45
Residual health complaints (*n* = 510; *n* = 23)				
Total	58	11	23	100
Sensory or musculoskeletal symptoms	43	8	20	87
Vision, hearing loss, tinnitus	17	3	7	30
Cognitive or mental symptoms	27	5	9	40

The *n* varies due to internal missing data

*ISCED 1997* International Standard Classification of Education 1997 (UNESCO-UIS 2006), *SD *standard deviation

Regarding their emotional response at the time of the accident, 28% of the respondents reported having felt incapacitated, 6% had experienced mortal fear, and 45% had experienced rage. Thirty-five percent reported having had health complaints (pain, muscular, sensory, cognitive or mental symptoms, or symptoms of the eyes or ears) after the incident. It was about twice as common to report physical symptoms compared to mental symptoms. There was an overlap between the two variables in that respondents often reported both physical and mental symptoms; five persons reported having only mental symptoms after the accident.

At the time of the survey, 11% (*n* = 58) reported residual health complaints (unchanged, decreased, or increased problems) that they attributed to EI (Table 1). These were later the base for recruitment to the clinical study. Of those with residual health complaints, the majority (74%) reported pain, muscular, or sensory symptoms, whereas 47% reported cognitive or mental symptoms, and 29% reported symptoms of the eyes or ears. For a more detailed description of the survey study group, see Rådman et al (2016b).

#### Self-reported mental wellbeing and cognitive problems

Compared to the reference data from a group of healthy subjects (*n* = 98) with similar demographics (Persson et al. 2002), the survey study electricians reported statistically significantly higher values on the SCL subscales Somatization and Anxiety (Table 2). Further, the survey study group reported more problems with attention in the EQ-9, compared to the reference data.

**Table 2:** Symptom Checklist-90 (SCL-90) and Euroquest-9 (EQ-9) subscales in the survey study group vs. reference data; results of *t *tests

	Survey study group		Reference data		*p* value
	*N*	M (SD)	*n*	M (SD)	
SCL subscales	507		98		
Somatization		0.47 (0.48)		0.30 (0.30)	< 0.001
Anxiety		0.38 (0.46)		0.24 (0.30)	< 0.001
Depression		0.39 (0.47)		0.30 (0.41)	0.11
EQ-9 subscales	505		98		
Memory		1.63 (0.50)		1.58 (0.41)	0.27
Attention		1.42 (0.48)		1.31 (0.34)	< 0.01
Total		1.56 (0.45)		1.49 (0.35)	0.07

*M* mean, *SD* standard deviation

#### Associations between independent variables and subjective psychological and cognitive outcomes

Regression analyses were carried out with the independent variables (circumstances of the accident, emotional responses, and health complaints after the accident) and using the SCL subscales and the EQ-9 as the outcomes, adjusted for age and time since the accident. Having been exposed to high (compared to low) voltage or a no-let-go situation was not statically significantly associated with any of the outcomes, while if the current pathway had been bilateral was associated with higher scores in SCL Somatization (Table 3). Having felt dazed at the time of the accident was associated with higher scores in SCL Somatization and SCL Anxiety, and had a borderline association with higher scores in EQ Attention. Loss of consciousness was associated with higher scores in SCL Somatization, EQ Memory, and the total EQ-9 score. To have felt incapacitated at the time of the accident was associated with higher scores in all outcomes (but a borderline association with EQ Memory), and to have experienced mortal fear was clearly associated with higher scores in all outcomes. To have experienced rage was associated with higher scores in all three SCL subscales, but not in the EQ-9. Reporting health complaints lasting > 1 week after the accident was associated with higher scores in all outcomes, whereas health complaints lasting < 1 week was associated with higher scores in SCL Somatization only. When separating physical and mental health complaints, the risk estimates for mental symptoms were elevated relative to those for physical symptoms, and they were more consistently statistically significant. It was enough to have had mental symptoms less than a week after the accident to have higher scores in EQ Memory and the total EQ-9.

**Table 3:** Linear regressions of independent variables (circumstances of the accident, emotional response, and health complaints after the accident) in relation to psychological problems [Symptom Checklist-90 (SCL-90) subscales] and subjective cognitive problems [Euroquest-9 (EQ-9) subscales]

	SCL SOM		SCL ANX		SCL DEP		EQ-9 MEM		EQ-9 ATT		EQ-9 TOT	
	est.	*p*	est.	*p*	est.	*p*	est.	*p*	est.	*p*	est.	*p*
Voltage												
> 1000 V	0.13	0.29	− 0.08	0.54	− 0.02	0.88	0.18	0.16	0.17	0.16	0.17	0.13
< 1000 V	ref		ref		ref		ref		ref		ref	
Pathway												
Bilateral	0.14	*	0.09	0.11	0.10	0.06	0.10	0.11	0.01	0.81	0.07	0.20
Unilateral	ref		ref		ref		ref		ref		ref	
No-let-go												
Yes	0.11	0.06	0.06	0.35	0.07	0.24	0.04	0.50	0.05	0.37	0.04	0.41
No	ref		ref		ref		ref		ref		ref	
Dazed												
Yes	0.15	**	0.12	*	0.08	0.17	0.09	0.12	0.11	0.05	0.10	0.07
No	ref		ref		ref		ref		ref		ref	
Unconscious												
Yes	0.27	**	0.08	0.44	.10	0.31	0.22	*	0.19	0.07	0.21	*
No	ref		ref		ref		ref		ref		ref	
Incapacitated												
Yes	0.21	***	0.19	**	0.18	**	0.12	0.05	0.17	**	0.14	*
No	ref		ref		ref		ref		ref		ref	
Mortal fear												
Yes	0.51	***	0.54	***	0.52	***	0.47	***	0.49	***	0.48	***
No	ref		ref		ref		ref		ref		ref	
Rage												
Yes	0.08	0.14	0.12	*	0.11	*	0.08	0.15	0.06	0.25	0.07	0.15
No	ref		ref		ref		ref		ref		ref	
Health complaints												
> 1 week	0.36	***	0.31	***	0.30	***	0.36	***	0.30	***	0.34	***
< 1 week	0.15	*	0.10	0.12	0.05	0.39	0.07	0.29	0.01	0.86	0.05	0.40
No	ref		ref		ref		ref		ref		ref	
Physical symptoms												
> 1 week	0.27	***	0.12	0.14	0.14	0.07	0.30	***	0.22	**	0.28	***
< 1 week	0.18	**	0.14	*	0.10	0.14	0.07	0.26	0.04	0.52	0.06	0.30
No	ref		ref		ref		ref		ref			
Mental symptoms												
> 1 week	0.73	***	0.85	***	0.86	***	0.62	***	0.69	***	0.65	***
< 1 week	− 0.02	0.76	0.09	0.26	0.00	0.99	0.17	*	0.10	0.23	0.15	*
No	ref		ref		ref		ref		ref		ref	

All analyses were adjusted for age and time since the accident. The *n* varied between 283 and 369, due to internal missing data. Unstandardized regression coefficient estimates (est.) and *p *values are shown

*ATT* attention, *ANX* anxiety, *DEP *depression, *MEM* memory, *ref* referent value, *SOM* somatization, *TOT* total

*p < 0.05; **p < 0.01; ***p < 0.001

In a second model (not presented in a table), the variable Health complaints after the accident were added to all analyses, in addition to age, and time since the accident. Having experienced mortal fear at the time of the accident remained associated with higher scores in all outcomes (with estimates varying from 0.37 to 0.44). In addition, having felt rage at the time of the accident remained associated with higher scores in SCL Anxiety and SCL Depression (estimates of 0.14). Bilateral current pathway, and having felt incapacitated, remained associated with higher scores in SCL Somatization (estimates of 0.12). Having felt incapacitated also had a borderline association with SCL Depression (estimate of 0.12). Loss of consciousness and having felt dazed no longer had statistical significance. In this second model, health complaints > 1 week remained a risk factor for all outcomes in all analyses, and health complaints < 1 week remained a risk factor for SCL Somatization in about half of the analyses.

### Clinical study

#### Descriptives

The clinical study group contained 23 male electricians 25–68 years of age who had all reported residual health complaints attributed to EI (Table 1). Their mean age was greater by 5 years than that of the survey study group, while the groups’ educational level was about the same. A slight majority indicated that they had experienced only one severe episode of electrical current passing through the body. Most of the remaining participants indicated between two and ten episodes and two participants had had more than 20 episodes. The subjectively perceived most severe incident had occurred between less than 6 months and almost 44 (median 5.5) years prior to the survey. The most common contact point for the current was the hand or finger. Three participants (13%) indicated that the head had been contact point for the current (data not shown). Most participants (82%) had felt dazed after the incident, and eight participants (36%) reported that they had lost consciousness.

A comparison of those who participated in the clinical study (*n* = 23) and those who were eligible but did not participate (*n* = 31) showed no statistically significant differences in demographics, accident reports, or reported health complaints. However, there was a tendency, though not statistically significant, for the clinical study participants to more often have had high voltage accidents, as well as more often reporting sensory and muscular symptoms and less often reporting cognitive or mood symptoms, than those who did not participate.

#### Self-reported cognitive problems and mental wellbeing

The clinical study group reported more cognitive problems than the matched control group, with a higher total score of the EQ-9 as well as in the two subscales EQ Memory and EQ Attention (Table 4)*.* Furthermore, the clinical study group reported lower mental wellbeing than the control group, with higher scores in SCL Somatization and SCL Anxiety, and a tendency for higher SCL Depression scores (Table 4).

**Table 4:** Symptom Checklist-90 (SCL-90), Euroquest-9 (EQ-9), and cognitive test results for the clinical study group (*n* = 23) and the matched control group (*n* = 23)

	Clinical study group *n* = 23		Matched control group *n* = 23		*p value*
	M	SD	M	SD	
SCL subscales					
Somatization	0.92	0.67	0.30	0.36	< 0.001^a^
Anxiety	0.65	0.85	0.22	0.24	0.034^a^
Depression	0.65	0.78	0.34	0.40	0.081^a^
EQ-9 subscales					
Memory	1.96	0.64	1.58	0.42	0.022^b^
Attention	1.71	0.67	1.29	0.38	0.011^a^
Total	1.87	0.62	1.48	0.38	0.013^b^
Cognitive tests					
Premorbid intellectual capacity					
SRB:1 synonyms	20.5	4.0	21.7	5.8	0.063^a^
WAIS-R information	22.2	3.4	21.6	4.0	0.40^a^
Visuospatial construction					
WAIS-R block design	35.6	7.2	32.0	9.9	0.17^b^
Short-term memory (episodic memory)					
Cronholm-Molander word pairs, immediate	19.4	6.7	20.3	4.8	0.62^b^
Cronholm-Molander word pairs, delayed	15.2	6.8	14.9	4.6	0.88^b^
* Austin Maze test, errors*	*25.7*	*15.4*	*35.3*	*22.1*	0.17^a^
* Austin Maze test, exeution time (s)*	*247.9*	*88.9*	*343.7*	*164.1*	0.052^a^
Attention/concentration/working memory					
WAIS-R digit symbol	48.6	8.9	50.0	10.6	0.61^b^
d2 test of attention, TN-E	369.0^c^	50.5	–	–	–
d2 test of attention, CP	145.6^d^	20.4	–	–	–
DS incidental learning, correct	6.8	1.9	5.7^e^	2.4^e^	0.054^a^
* DS incidental learning, errors*	*0.8*	*1.3*	*2.1*^e^	*1.8*^e^	0.004^a^
F-A-S verbal fluency	39.6	9.5	38.4	12.0	0.72^b^

For the SCL and EQ-9 subscales, higher scores indicate a higher degree of subjective problems. For the cognitive tests, higher scores indicate better performance, except for test variables in *italics*, where lower scores indicate better performance

*M* mean, *SD* standard deviation

^a^Mann–Whitney *U* test

^b^*t *test

^c,^^d^Only official normative data were used (Brickenkamp and Zillmer 1998); the group mean for TN-E corresponds to the 62nd percentile for the age group 40–49 years, and that for CP to somewhat above the 50th percentile (same age group)

^e^Only data from a demographically different reference group were available (*n* = 50, mean age 50 years, 74% women, 72% university-educated) (Österberg et al. 2014)

#### Cognitive tests

The only statistically significant differences in test scores between the clinical study group and the matched control group were in execution time on the Austin Maze memory test, and in the number of errors on the Digit Symbol subtest for Incidental Learning (Table 4). These differences, however, were in favor of the clinical study group: the electricians solved the memory task faster and showed better incidental learning compared to the matched controls. A tendency for a difference between the groups regarding the verbal skills test (SRB:1) was also seen (*p* = 0.063), this time with better results for the control group. The SRB:1, which is normally considered fairly insensitive to mild to moderate encephalopathy, was intended to reflect premorbid cognitive function. Therefore, the tendency towards better verbal skills in the control group primarily confirms the view that this group was at least on a par with the clinical study group regarding general intellectual level.

## Discussion

The electricians in the survey study (*n* = 510), who had all experienced at least one incident with electrical current passing through the body, reported a higher degree of problems with attention and more psychological problems in terms of somatization and anxiety, compared to reference data. This is in line with several previous studies that report reduced cognitive functions and poorer mental wellbeing after EI (e.g., Kelley et al. 1999; Piotrowski et al. 2014; Pliskin et al. 1998; Ramati et al. 2009; Singerman et al. 2008). A strength of the current study is that it examined a relatively unselected group, i.e., not patients seeking treatment for cognitive or psychiatric impairment in the sequelae of an electrical accident. Only about 25% of the respondents had sought medical care after the accident (Rådman et al. 2016b). However, in contrast to the reported subjective cognitive problems, we found no objectively verifiable reduction in cognitive function in the clinical examinations of a subgroup of electricians. There was no evidence of lower performance in a range of tests for short-term memory, visuospatial construction, attention, concentration, and working memory. Rather, there was occasional evidence of better performance among the electricians compared to the control group. This was in spite of the finding that they reported more subjective problems with attention and memory than the matched controls, that they had experienced fairly severe accidents, with the vast majority having felt dazed in the aftermath and many of them having had at least a brief loss of consciousness, and in addition, that they had all reported residual health complaints that they attributed to EI.

It is difficult to give a clear interpretation of the discrepancy between the absence of objective cognitive reduction and the presence of subjective cognitive problems; several explanations are possible. For example, the neuropsychological tests conducted may have been insufficiently susceptible to cognitive reduction due to EI. However, we believe that the cognitive domains considered sensitive to EI, i.e., learning and memory, attention, and cognitive speed (Duff and McCaffrey 2001; Pliskin et al. 1998, 2006), were covered by the tests. In addition, several of the selected tests have demonstrated high sensitivity for mild cognitive impairment in other subtle organic conditions, such as chronic toxic encephalopathy (Österberg et al. 2000) and exhaustion disorder/burnout (Österberg et al. 2012). Another possible explanation is that the control group was not a sufficient match to the clinical study group, although they were matched by sex and age, educational level, and type of occupation. For a few test–score parameter comparisons used in the clinical study, optimal control group data were not available. For these, we used either a highly educated reference group (which also included women), or official normative data for the test, which comprised a general population sample (including both women and men). It would have been optimal had the control group been a group of uninjured electricians, but this was not feasible within the current study design. The matched control group tended to perform better on the verbal skills test that is often used as a control for good matching (as learned verbal skills are considered resistant to mild to moderate diffuse brain damage). From this perspective, this would indicate that the controls were slightly more cognitively capable than the electricians. Which, together with that a few comparisons, used reference data from a highly educated group, should have increased the likelihood to detect group differences to the electricians’ disadvantage. Despite this, no group differences in this direction were detected in the results. Another potential source of bias was that we used existing data from a previous control group, and that test administration effects may have influenced results.

The relatively small group size of the clinical study implies lacking statistical power to detect group differences. However, with the trend towards better performance of the clinical study group in some tests, it seems unlikely that a bigger group size would have resulted in statistically significant lower performance of the clinical group—unless there was bias in the selection of participants. It is possible that there is a significant number of unknown cases among those eligible individuals lost to participation. Of the 54 who were eligible for the clinical study, only 23 participated. Apart from the fact that some were excluded for health reasons, it is conceivable that cognitive difficulties could have been an underlying reason for refraining from participation. If this was the case, this study may convey a false impression that EI does not lead to cognitive effects. Consequently, it should be emphasized that the results may not necessarily be generalizable to a population of individuals seriously affected by EI. In other words, the results do not disaffirm that patients presenting with symptoms may have cognitive dysfunction as an effect of EI. The good news is that exposure to electrical current passing through the body does not seem to inevitably lead to measureable cognitive dysfunction.

The mechanisms for long-term effects on mood and cognition are not clear. Andrews and Reisner (2017) discuss several potential mechanisms for long-term and/or delayed neuropsychiatric symptoms after EI, including the release of neuroactive substances such as cortisol. The mechanisms may be multiple; the severity of the accident, physical aspects including the current’s path in the body, together with the degree of vulnerability of the individual, may interplay with how the individual is psychologically able to process a potentially fatal accident. An obvious risk factor for later subjective psychological and cognitive problems in our study was having had health complaints for > 1 week after the accident, especially if these included mental symptoms. About 10% of the survey participants, and all those who participated in the clinical study, reported residual health complaints that they attributed to EI, mostly sensory and musculoskeletal disorders. It is quite likely that the elevated SCL Somatization scores were due to actual health problems. However, there was also a higher degree of reported anxiety among the electricians compared to the referents. A possible cause of long-term reduced mental wellbeing is the psychological trauma of having had a life-threatening accident, which can manifest as PTSD or increased anxiety or depression. Psychiatric problems, such as anxiety, depression, phobic reactions, and PTSD appear to be fairly prevalent after electrical accidents (Kelley et al. 1999; Piotrowski et al. 2014; Pliskin et al. 1998; Primeau et al. 1995; Ramati et al. 2009). However, the level of problems seems difficult to predict. For example, in Piotrowski et al. (2014), there were no significant correlations between injury severity and PTSD in electrically injured workers. In our study, neither voltage level nor having experienced the no-let-go phenomenon was statistically significantly associated with later subjective cognitive or psychological problems (although there were elevated risk estimates). However, it is quite possible that our study lacked power to detect voltage as a risk factor due to the low number of participants (*n* = 18) with high voltage accidents. While voltage level has been seen to be unrelated to cognitive and mental outcomes also in other studies (e.g., Bailey et al. 2008; Ramati et al. 2009), we were rather surprised that the no-let-go phenomenon did not appear as a (statistically significant) risk factor. The no-let-go phenomenon emerged as particularly stressful in the interviews with the electricians in the clinical study, and was described as having induced severe anxiety and mortal fear (Thomée and Jakobsson 2018). In addition, it was reported as a risk factor for PTSD among EI patients in Kelley et al. (1999).

Besides the previously mentioned methodological issues regarding the clinical study, several other limitations need to be addressed. For example, the time that had passed since the accident varied from less than 1 year to almost 45 years, and it may obviously be difficult to remember all aspects of an accident after many years, even decades. The validity of these retrospective responses can be questioned. Recall bias may be present in that those with currently reduced wellbeing may remember the accident in negative terms and emphasize certain aspects such as the emotional response or health complaints after the accident. Correspondingly, those with no long-term health consequences may remember the accident in not such negative terms. If this is the case, the associations between circumstances of the accident and later subjective problems may be overestimated. It should also be noted that the rather large variation in reported number of severe incidents (from 0 to 90) in the survey study, may indicate a differential threshold for reporting an incident to be severe among the electricians. The severity of the most severe incident evidently varied, which was illustrated in the interview study of the clinical study participants (Thomée and Jakobsson 2018). For some, the most severe incident was a very brief shock and for others a life-threatening event leading to long-term hospitalization. However, this means that the study included the broad variety of incidents that professional electricians may encounter, as intended. Nonetheless, including both high voltage and low voltage accidents in the study has the potential to blur results. In the supplementary stratification of the survey data, the high voltage group more often reported having lost consciousness at the time of the accident and health complaints in the aftermath of the accident, as well as residual health problems attributed to electrical injury. However, the high voltage group was small (*n* = 18) and does not seem to have an impact on the main results. In the supplementary regression analyses in the low voltage group, a few associations lost statistical significance, but the main results remained intact, i.e., having experienced mortal fear at the time of the accident and/or health complaints > 1 week, especially if these included mental symptoms, were risk factors for all outcomes of later reduced mental well-being and subjective cognitive function. The estimates regarding mental symptoms as a risk factor were even higher in the low voltage group than in the total study group.

Another important limitation is loss to participation. Although the participants were fairly unselected compared to studies that are based on patient populations, the participation rate was only about 50% in all steps of inclusion to the studies. Therefore, selection bias may have affected generalizability of the results. It is possible that those who participated were more concerned about health issues than those who did not participate, and this may have amplified the results regarding levels of subjective psychological and cognitive problems. However, as mentioned previously, with regard to the clinical study, it is also possible that some individuals with reduced cognitive abilities declined participation, which would imply an underestimation of cognitive dysfunction in the clinical study.

Another limitation which affects generalizability is that only professional electricians were included in the study, and these generally account for less than half of all reported electrical accidents leading to sick leave in Sweden (Elsäkerhetsverket 2017). While electricians are experts in their field and have knowledge about the risks of working with electricity, it is possible that other professionals and non-professionals will react psychologically differently to an electrical accident. In addition, only male electricians were included, and therefore, the results may not be transferable to women.

It has been suggested that being a professional electrician may increase the vulnerability to develop depression after an accident, due to the loss of self-perception as a competent electrical worker (Kelley et al. 1999). Further, in the field of professional electricians, there are most likely masculinity norms at play (Stergiou-Kita et al. 2015, 2016). These norms, which favor toughness and risk acceptance, may also de-legitimize the severity of injury (Stergiou-Kita et al. 2016), and consequently reduce the victim’s inclination to seek medical or psychological help. Interestingly, in a survey of electricians, the majority of the respondents reported thinking about EI on a daily basis, but speaking about it more rarely (Tkachenko et al. 1999). Most of the electricians never or rarely spoke with family members about these concerns (Tkachenko et al. 1999). This implies a self-inflicted lack of access to social support to process thoughts about EI and potential health effects.

It seems important to raise awareness of the potential long-term consequences of EI, both in the electrical trade and in the health care services. A certain lack of knowledge in the health care system, and the difficulties to predict later problems from initial damage, can lead to some controversy when the EI victim seeks medical attention (Primeau 2005; Primeau et al. 1995). If an EI victim does not present clear tissue damage, monitoring or follow-ups are uncommon. Andrews (2012) points out that psychological disability may be the biggest problem for an EI patient. While physical disabilities are often accommodated in a rehabilitation process after EI, cognitive and psychological problems can be a challenge for successful return to work (Stergiou-Kita et al. 2014a, b; Theman et al. 2008). Andrews et al. (2017) even propose including a specific post-electrical and post-lightning injury syndrome in the American Psychiatric Association’s *Diagnostic and Statistical Manual* (*DSM*), with diagnostic criteria that include neuropsychiatric aspects such as cognitive dysfunction and psychiatric symptoms. They argue that the diagnostic criteria can be an instrument for assessment of the patients; also, they can facilitate future systematic research on the topic.

## Conclusions

In this study of electricians who had experienced at least one occupational accident involving electrical current passing through the body, the participants reported more subjective cognitive problems and lower mental wellbeing compared to reference data, but no long-term objective cognitive dysfunction was detected. Emotional response at the time of the accident and health complaints, especially mental symptoms, in the aftermath of the accident may constitute important indications for medical and psychological follow-ups.

## Electronic supplementary material

Below is the link to the electronic supplementary material.

Supplementary file1 (DOCX 29 kb)
