# Supplementary material for: Cognition and mental wellbeing after electrical accidents: a survey and a clinical study among Swedish male electricians
Source: Int Arch Occup Environ Health. 2020 Feb 8;93(6):683–96. doi: 10.1007/s00420-020-01520-x (PMC7320954; doi:10.1007/s00420-020-01520-x)
Supplement: Supplementary file 1 — Supplementary file1 (DOCX 29 kb) [file 420_2020_1520_MOESM1_ESM.docx]

Supplementary Table 5. Descriptive statistics of the survey study group stratified for reported voltage of the most severe incident. The *n* varies due to internal missing data.

|  |  | Low voltage  n=420 | |  | High voltage  n=18 | |  |
| --- | --- | --- | --- | --- | --- | --- | --- |
| Age, yrs |  |  | |  |  | |  |
| Mean (SD) |  | 42.9 (12.9) | |  | 46.0 (12.6) | |  |
| Median |  | 43.0 | |  | 45.5 | |  |
| Range |  | 20-66 | |  | 28-68 | |  |
| No. of severe electrical shocks (n=398; n=17) |  |  | |  |  | |  |
| Mean (SD) |  | 3.8 (6.5) | |  | 2.1 (2.2) | |  |
| Median |  | 2.0 | |  | 1.0 | |  |
| Range |  | 0-90 | |  | 1-10 | |  |
| No. of years since the most severe accident (n=348; n=18) |  |  | |  |  | |  |
| Mean (SD) |  | 11.1 (10.5) | |  | 5.3 (4.5) | |  |
| Median |  | 6.9 | |  | 4.3 | |  |
| Range |  | 0-44.7 | |  | 0.4-15.7 | |  |
|  |  | n | % |  | n | % |  |
| Education level (ISCED 1997) |  |  |  |  |  |  |  |
| Primary school (level 2) |  | 28 | 7 |  | 3 | 17 |  |
| Secondary school (level 3) |  | 362 | 87 |  | 13 | 72 |  |
| University/college (level 5) |  | 25 | 6 |  | 2 | 11 |  |
| Circumstances of the most severe incident |  |  |  |  |  |  |  |
| Voltage |  |  |  |  |  |  |  |
| ≤1000 V |  | 420 | 100 |  | - | - |  |
| >1000 V |  | - | - |  | 18 | 100 |  |
| Electrical current contact points |  |  |  |  |  |  |  |
| Hand – hand |  | 213 | 52 |  | 5 | 31 |  |
| Hand – other/unknown |  | 177 | 43 |  | 9 | 56 |  |
| Other – other/unknown |  | 19 | 5 |  | 2 | 12 |  |
| Current pathway |  |  |  |  |  |  |  |
| Bilateral |  | 158 | 50 |  | 7 | 64 |  |
| Unilateral |  | 158 | 50 |  | 4 | 36 |  |
| No-let-go |  |  |  |  |  |  |  |
| Yes |  | 107 | 26 |  | 6 | 33 |  |
| No |  | 310 | 74 |  | 12 | 67 |  |
| Loss of consciousness |  |  |  |  |  |  |  |
| Yes |  | 15 | 4 |  | 9 | 53 |  |
| No |  | 374 | 94 |  | 8 | 47 |  |
| Uncertain |  | 10 | 3 |  | - | - |  |
| Dazed |  |  |  |  |  |  |  |
| Yes |  | 254 | 62 |  | 13 | 76 |  |
| No |  | 142 | 35 |  | 2 | 12 |  |
| Uncertain |  | 13 | 3 |  | 2 | 12 |  |
| Emotional response at the time of the accident |  |  |  |  |  |  |  |
| Incapacitated |  |  |  |  |  |  |  |
| Yes |  | 113 | 28 |  | 4 | 25 |  |
| No |  | 283 | 69 |  | 9 | 56 |  |
| Uncertain |  | 13 | 3 |  | 3 | 19 |  |
| Mortal fear |  |  |  |  |  |  |  |
| Yes |  | 20 | 5 |  | 3 | 19 |  |
| No |  | 377 | 94 |  | 10 | 62 |  |
| Uncertain |  | 6 | 1 |  | 3 | 19 |  |
| Rage |  |  |  |  |  |  |  |
| Yes |  | 185 | 45 |  | 5 | 29 |  |
| No |  | 209 | 51 |  | 10 | 59 |  |
| Uncertain |  | 14 | 3 |  | 2 | 12 |  |
| Health complaints attributed to electrical injury |  |  |  |  |  |  |  |
| Yes, >1 week after the accident |  | 52 | 13 |  | 11 | 65 |  |
| Yes, <1 week after the accident |  | 87 | 21 |  | 1 | 6 |  |
| No |  | 267 | 66 |  | 5 | 29 |  |
| Physical symptoms |  |  |  |  |  |  |  |
| Yes, >1 week after the accident |  | 43 | 11 |  | 10 | 59 |  |
| Yes, <1 week after the accident |  | 88 | 22 |  | 1 | 6 |  |
| No |  | 275 | 68 |  | 6 | 35 |  |
| Mental symptoms |  |  |  |  |  |  |  |
| Yes, >1 week after the accident |  | 17 | 4 |  | 6 | 38 |  |
| Yes, <1 week after the accident |  | 44 | 11 |  | 2 | 12 |  |
| No |  | 344 | 85 |  | 8 | 50 |  |
| Residual health complaints (n=420; n=18) |  |  |  |  |  |  |  |
| Total |  | 46 | 11 |  | 10 | 56 |  |
| Sensory or musculoskeletal symptoms |  | 31 | 7 |  | 10 | 56 |  |
| Vision, hearing loss, tinnitus |  | 14 | 3 |  | 2 | 11 |  |
| Cognitive or mental symptoms |  | 21 | 5 |  | 6 | 33 |  |

ISCED 1997 = International Standard Classification of Education 1997 (UNESCO-UIS 2006). SD = standard deviation.

Supplementary Table 6. Symptom Checklist-90 (SCL-90) and Euroquest-9 (EQ-9) subscales in the survey study group stratified for reported voltage of the most severe incident.

|  |  | Low voltage | |  | High voltage | |  |
| --- | --- | --- | --- | --- | --- | --- | --- |
|  |  | n | M (SD) |  | n | M (SD) |  |
| SCL subscales |  | 419 |  |  | 18 |  |  |
| Somatization |  |  | 0.49 (0.49) |  |  | 0.62 (0.66) |  |
| Anxiety |  |  | 0.41 (0.48) |  |  | 0.36 (0.49) |  |
| Depression |  |  | 0.40 (0.47) |  |  | 0.41 (0.57) |  |
| EQ-9 subscales |  | 415 |  |  | 18 |  |  |
| Memory |  |  | 1.64 (0.50) |  |  | 1.79 (0.71) |  |
| Attention |  |  | 1.43 (0.47) |  |  | 1.59 (0.76) |  |
| Total |  |  | 1.57 (0.45) |  |  | 1.72 (0.71) |  |

M = mean; SD = standard deviation.

Supplementary Table 7. Low voltage group (n=420): Linear regressions of independent variables (circumstances of the accident, emotional response, and health complaints after the accident) in relation to psychological problems (Symptom Checklist-90 (SCL-90) subscales) and subjective cognitive problems (Euroquest-9 (EQ-9) subscales). All analyses were adjusted for age and time since the accident. The *n* varied between 267 and 344, due to internal missing data. Unstandardized regression coefficient estimates (est.) and *p*-values are shown.

|  | SCL SOM | | SCL ANX | | SCL DEP | | EQ-9 MEM | | EQ-9 ATT | | EQ-9 TOT | |
| --- | --- | --- | --- | --- | --- | --- | --- | --- | --- | --- | --- | --- |
|  | est. | *p* | est. | *p* | est. | *p* | est. | *p* | est. | *p* | est. | *p* |
| No-let-go |  |  |  |  |  |  |  |  |  |  |  |  |
| Yes | 0.09 | 0.11 | 0.03 | 0.59 | 0.05 | 0.43 | 0.02 | 0.73 | 0.04 | 0.52 | 0.03 | 0.62 |
| No | ref |  | ref |  | ref |  | ref |  | ref |  | ref |  |
| Pathway |  |  |  |  |  |  |  |  |  |  |  |  |
| Bilateral | 0.12 | * | 0.09 | 0.13 | 0.08 | 0.13 | 0.07 | 0.27 | -0.01 | 0.91 | 0.04 | 0.44 |
| Unilateral | ref |  | ref |  | ref |  | ref |  | ref |  | ref |  |
| Dazed |  |  |  |  |  |  |  |  |  |  |  |  |
| Yes | 0.14 | * | 0.11 | 0.06 | 0.06 | 0.28 | 0.06 | 0.27 | 0.08 | 0.16 | 0.07 | 0.19 |
| No | ref |  | ref |  | ref |  | ref |  | ref |  | ref |  |
| Unconscious |  |  |  |  |  |  |  |  |  |  |  |  |
| Yes | 0.30 | * | 0.15 | 0.28 | 0.16 | 0.21 | 0.16 | 0.21 | 0.20 | 0.12 | 0.18 | 0.14 |
| No | ref |  | ref |  | ref |  | ref |  | ref |  | ref |  |
| Incapacitated |  |  |  |  |  |  |  |  |  |  |  |  |
| Yes | 0.19 | ** | 0.18 | ** | 0.16 | ** | 0.10 | 0.11 | 0.15 | * | 0.11 | * |
| No | ref |  | ref |  | ref |  | ref |  | ref |  | ref |  |
| Mortal fear |  |  |  |  |  |  |  |  |  |  |  |  |
| Yes | 0.44 | *** | 0.48 | *** | 0.44 | *** | 0.42 | *** | 0.39 | *** | 0.41 | *** |
| No | ref |  | ref |  | ref |  | ref |  | ref |  | ref |  |
| Rage |  |  |  |  |  |  |  |  |  |  |  |  |
| Yes | 0.06 | 0.27 | 0.12 | * | 0.09 | 0.07 | 0.06 | 0.25 | 0.05 | 0.37 | 0.06 | 0.26 |
| No | ref |  | ref |  | ref |  | ref |  | ref |  | ref |  |
| Health complaints |  |  |  |  |  |  |  |  |  |  |  |  |
| >1 week | 0.37 | *** | 0.36 | *** | 0.32 | *** | 0.42 | *** | 0.35 | *** | 0.40 | *** |
| <1 week | 0.16 | ** | 0.11 | 0.10 | 0.06 | 0.32 | 0.08 | 0.20 | 0.03 | 0.68 | 0.06 | 0.27 |
| No | ref |  | ref |  | ref |  | ref |  | ref |  | ref |  |
| Physical symptoms |  |  |  |  |  |  |  |  |  |  |  |  |
| >1 week | 0.27 | ** | 0.16 | 0.07 | 0.16 | 0.06 | 0.34 | *** | 0.25 | ** | 0.31 | *** |
| <1 week | 0.19 | ** | 0.12 | 0.07 | 0.08 | 0.22 | 0.08 | 0.22 | 0.04 | 0.56 | 0.07 | 0.27 |
| No | ref |  | ref |  | ref |  | Ref |  | ref |  | ref |  |
| Mental symptoms |  |  |  |  |  |  |  |  |  |  |  |  |
| >1 week | 0.92 | *** | 1.13 | *** | 1.09 | *** | 0.76 | *** | 0.83 | *** | 0.78 | *** |
| <1 week | -0.02 | 0.79 | 0.09 | 0.26 | 0.00 | 0.96 | 0.20 | * | 0.11 | 0.16 | 0.17 | * |
| No | ref |  | ref |  | ref |  | ref |  | ref |  | ref |  |

ATT = attention; ANX = anxiety; DEP = depression; MEM = memory; ref = referent value; SOM = somatization; TOT = total.
*p<0.05; **p<0.01; ***p<0.001.
